# Cyber-Sexual Crime and Social Inequality: Exploring Socioeconomic and Technological Determinants

**DOI:** 10.3390/bs15111547

**Published:** 2025-11-13

**Authors:** Carlos J. Mármol, Aurelio Luna, Isabel Legaz

**Affiliations:** Department of Legal and Forensic Medicine, Biomedical Research Institute of Murcia (IMIB), Regional Campus of International Excellence “Campus Mare Nostrum”, Faculty of Medicine, University of Murcia (UMU), 30100 Murcia, Spain; cj.marmol@um.es (C.J.M.); aurluna@um.es (A.L.)

**Keywords:** cyber-sexual crime, digital access, economics and Spanish law, online harassment, socioeconomic inequality

## Abstract

Cyber-sexual crimes have become a growing concern in the digital age, as rapid technological progress continues to create new forms of violence and victimization. These offenses affect society unevenly, striking more intensely among minors, women, and other vulnerable groups. Their prevalence is shaped by structural inequalities, educational, economic, and technological, that condition both exposure to digital risks and the capacity for protection. Although international research has connected these disparities with digital victimization, evidence from Spain remains limited. The aim was to analyze the regional distribution of cyber-sexual crimes in Spain between 2011 and 2022 and to explore how education, income, and digital access relate to their incidence. To this end, official data from the Spanish Statistical Crime Portal (PEC) were combined with structural indicators provided by the Spanish National Institute of Statistics. The analysis encompassed reported cases of sexual abuse, sexual harassment, corruption of minors, online grooming, exhibitionism, pornography, and sexual provocation, using standardized incidence rates per 100,000 inhabitants. Statistical methods included ANOVA with post hoc comparisons, correlation analyses, and K-means clustering to identify territorial patterns. Results revealed a sustained national increase in cyber-sexual crimes, with grooming and sexual harassment showing the most pronounced growth. The Balearic Islands (mean 4.9), Canary Islands (4.0), and Andalusia (3.9) registered the highest incidence rates, well above the national average (3.0). Educational disadvantages and low income were linked to sexual abuse and corruption of minors, whereas greater digital connectivity, expressed through higher mobile phone use, broadband access, and computer ownership, was strongly associated with grooming and other technology-facilitated offenses. Cluster analysis identified three distinct territorial profiles: high-incidence regions (Balearic and Canary Islands, Andalusia), intermediate (Murcia, Madrid, Navarre, Valencian Community), and low-incidence (Galicia, Catalonia, Castile and León, among others). In conclusion, the findings demonstrate that cyber-sexual crimes in Spain are unevenly distributed and closely linked to persistent structural vulnerabilities that shape digital exposure. These results underscore the need for territorially sensitive prevention strategies that reduce educational and economic inequalities, foster sexual and digital literacy, and promote safer online environments. Without addressing these underlying structural dimensions, public policies risk overlooking the conditions that sustain regional disparities and limit adequate protection against technology-driven sexual crimes.

## 1. Introduction

In the current socio-digital context, increasingly influenced by the pervasive presence of digital technologies, interactions across personal, professional, and social spheres are predominantly mediated through online platforms and digital devices (Thompson, 2020; Yadav & Pavlou, 2020). Although digitalization offers significant opportunities for communication and development (Secinaro et al., 2025), it has also coincided with the emergence of new forms of violence that are particularly complex and impactful (Abashidze & Goncharenko, 2022; Kostin et al., 2021).

Among these, cyber-sex crimes have emerged as a particularly concerning phenomenon due to their growing prevalence, multifaceted nature, and the long-term impact they generate on individuals and communities (Martellozzo, 2019; Policing Sex Crimes—Dale Spencer, Rosemary Ricciardelli—Google Libros, n.d.; Wall, 2015). These crimes encompass a broad spectrum of behaviors that involve violations of sexual privacy and consent facilitated by digital tools (Hedidi & Hedidi, 2023; Kumar, 2023; Powell, 2022). Common manifestations include the unauthorized dissemination of intimate images, covert recording of individuals’ bodies, sexual harassment within virtual environments, online grooming, sextortion, and the manipulation or fabrication of sexually explicit material using personal content (Harduf, 2020; Henry et al., 2020; Lee & Lee, 2024; Whittle et al., 2013). In this context, sextortion refers to making threats to share nude or sexual images to coerce the victim into complying with specific demands, such as paying a ransom, sharing intimate images, or engaging in unwanted acts (Ray & Henry, 2025). Rather than isolated incidents, cyber-sex crimes are increasingly understood as the product of structural and contextual factors that shape digital behavior and risk exposure. Studies showed that cyber-sexual crimes are widespread and shaped by structural inequalities (Henry et al., 2020; Henry & Powell, 2018). Evidence concerning children and adolescents is particularly alarming. In the United States, a nationally representative survey found that before age 18, 15.6% of respondents had experienced online child sexual abuse, 11.0% image-based abuse, 5.4% grooming, and 3.5% sextortion (Finkelhor et al., 2022). In the United Kingdom, reports indicate that 39% of children aged 8–17 have experienced bullying, primarily online, while ONS shows that 19.1% of children aged 10–15 experienced online bullying in the past year (Bullying and online experiences among children in England and wales: Year ending March 2023—Office for National Statistics, n.d.). In Spain, recent data show that 60.6% of young people have experienced some form of digital sexual violence, with the most frequent behaviors being the non-consensual receipt of sexual content (22.1%) and harassment based on physical appearance (21.3%) (Calderón Gómez et al., 2024). Research on adult populations demonstrates that these forms of cyber-sexual violence are not limited to minors. Other studies confirm that sexual minorities and younger adults are disproportionately targeted, while women report more severe psychosocial impacts (Umbach et al., 2025). Qualitative and legal scholarship highlights the gendered roots of image-based sexual abuse (McGlynn & Rackley, 2017), its severe emotional harms (McGlynn et al., 2021), and the fragmented institutional responses available to victims (Rackley et al., 2021). Comparable evidence from Australia also shows that technology-facilitated sexual violence is embedded in gender inequality, with cultural and legal contexts shaping both incidence and reporting (Henry et al., 2020; Henry & Powell, 2018). Taken together, this research indicates that cyber-sexual crimes are not evenly distributed but mediated by gender, sexuality, and broader structural conditions.

Despite the growing body of international evidence, comparative analyses addressing how these structural and sociodemographic variables operate within national contexts remain scarce. Spain lacks a systematic examination of territorial differences in the incidence of cyber-sexual crimes and their association with socioeconomic and digital indicators.

Therefore, the main research question guiding this study is: to what extent do regional socioeconomic, educational, and digital disparities explain the unequal distribution of cyber-sexual crimes across Spanish territories? By addressing this question, the study seeks to fill a significant empirical and analytical gap in the literature on technology-facilitated sexual violence, offering new insights into the structural determinants of these offenses. The findings may contribute to more context-sensitive prevention strategies, the design of evidence-based public policies, and a better understanding of how social inequalities shape digital victimization.

This study aims to examine the regional distribution of cyber-sexual crimes in Spain and to identify structural factors (such as educational attainment, income levels, and digital access) that may explain territorial disparities in offense rates.

## 2. Methods

### 2.1. Data on Sexual Cybercrime in Spain

The data used in this study were obtained from the Statistical Crime Portal (PEC) of the Spanish Ministry of the Interior and correspond to officially recorded cyber-sexual crimes in Spain (2011–2022) involving Spanish victims (Portal Estadístico de Criminalidad, n.d.). The term “crimes” is used according to the statistical definition applied by law enforcement, recognizing that police records capture reported and registered incidents which may not necessarily proceed to court or result in conviction.

The analysis focuses on the 2011–2022 period, when UNODC-led standardization enabled a consistent and comparable classification of Information and Communication Technologies (ICT)-related offenses in Spain (EU guidelines for the International Classification of Crime for Statistical Purposes-ICCS 2017 edition, n.d.; Monitoring EU crime policies using the International Classification of Crime for Statistical Purposes (ICCS), 2018).

To ensure comparability, incidence rates were calculated per 100,000 inhabitants using annual population estimates provided by the National Statistics Institute (INE) (Instituto Nacional de Estadística (INE), n.d.). Because the Mossos d’Esquadra (Catalan Police) and the Ertzaintza (Basque Police) began submitting data only in 2015, earlier figures from these regions may be incomplete. In addition to reported crime data, several structural and contextual variables (education, income, and digital access) were considered to explore territorial patterns.

### 2.2. Structure of the Spanish Educational System

The educational level was analyzed using data from the National Institute of Statistics (INE), disaggregated by autonomous community (Población de 16 y más años por nivel de formación alcanzado, sexo y comunidad autónoma. Porcentajes respecto del total de cada comunidad (6369), n.d.). For analytical purposes, these were grouped into three broader levels—low (up to primary), medium (secondary and vocational), and high (university or higher)—to ensure regional comparability and avoid over-fragmentation.

### 2.3. Income Levels, Digital Behavior, and Household Technological Equipment

Income level was also incorporated, based on classifications established by the INE’s Atlas of Household Income Distribution (Nota de Prensa: Atlas de Distribución de Renta de los Hogares. Año 2022, n.d.). These categories are defined according to the national median annual net income, which in 2022 stood at €18,316. Four groups were identified: low income (less than €13,737), lower-middle income (€13,737–€18,316), upper-middle income (€18,316–€36,632), and high income (above €36,632). We also included Internet and social media data to account for digital behavior patterns. These data were drawn from the INE’s ICT Survey on Households for 2022 (Instituto Nacional de Estadística (INE), n.d.). Two complementary indicators were analyzed: daily internet use (access on five or more days per week) and multiple daily internet use (several connections per day). Both serve as proxies for the intensity and frequency of online activity across Spain’s regions. Lastly, household access to technological equipment was examined using Spanish national data from the INE (Evolución de datos de Viviendas (2006–2024) por tamaño del hogar, hábitat, tipo de equipamiento y periodo, n.d.). This included the percentages of homes with a computer, internet access, a broadband connection, a landline, and a mobile phone. Unless otherwise specified, all values refer to the year 2022. Computers were defined as the availability of at least one device in the household, either a desktop or laptop, considered part of digital access resources. Internet Access refers to the actual ability of the household to connect to the network and use online services, regardless of the type of connection employed. In this regard, the survey explicitly distinguishes broadband as a specific type of technical connection, characterized by high speed and data transmission capacity, which may be provided through ADSL, fiber optic, cable, or other fixed-access technologies.

Additionally, the variable landline captures the availability of a fixed telephone line in the household, which in the original dataset was considered part of household ICT infrastructure. The variable mobile indicates the presence of at least one such device in the household, regardless of whether it includes internet access via mobile data. The Spanish Penal Code (notably Articles 183 and 189 bis) explicitly recognizes the telephone as a means of committing certain sexual offenses, without distinguishing between landlines or mobile phones. Consequently, offenses committed via telephone are included in the Ministry of Interior’s statistics on cyber-sexual crimes. Similar references appear in other offenses, including incitement of minors to self-harm, suicide, or drug use, as well as in threats. All these indicators were obtained from INE as relative population distributions (percentages of individuals or households in each category) and were incorporated as contextual variables. They were then statistically correlated with cyber-sexual crime incidence rates (per 100,000 inhabitants) at the regional level within an ecological framework.

### 2.4. Classification of Cyber-Sex Crimes and Victim Age Criteria

The cyber-sex crimes analyzed in this study follow the classification of the Statistical Crime Portal (PEC) of the Spanish Ministry of the Interior and correspond to officially recorded cases of cyber-sexual offenses (Data—Crime Statistics Portal, n.d.). These include: sexual abuse, referring to acts of a sexual nature carried out without consent, regardless of the victim’s age; sexual harassment, defined as unwanted sexual conduct, requests, or behavior that creates an intimidating, hostile, or humiliating environment, including in digital contexts; corruption of minors or persons with disabilities, which comprises any act that induces, facilitates, or promotes sexual behavior in individuals under 18 years of age or in persons with disabilities (Spanish Penal Code, Art. 183) (Artículo 183 Del Código Penal | Codigo Penal Español, n.d.); grooming, understood as online contact for sexual purposes, usually with the aim of gaining trust to later commit abuse or exploitation; exhibitionism, which involves exposing one’s body or performing obscene acts in the presence of others through online environments; pornography, covering the production, distribution, possession, or access to material that depicts sexual activity; and sexual provocation, which refers to the dissemination, sale, or public display of pornographic material through digital channels, with the purpose or effect of harming sexual freedom or development. All reported cases analysed in this study involved victims ranging from under 18 to those aged 65 or older.

### 2.5. Temporal Trend Analysis

To contextualize the regional and structural findings, a general trend analysis was conducted using simple linear regression models applied to the national incidence rates of cyber-sexual crimes. Annual standardized rates per 100,000 inhabitants were the dependent variable, with calendar year as the independent variable. Specific offense-level trends were analyzed descriptively.

### 2.6. Cluster Analysis of Sexual Crime Rates Across Spanish Regions

To classify the autonomous communities according to their mean sexual crime rates between 2011 and 2022, a cluster analysis was performed using the K-means algorithm (Bock, 2007). The optimal number of clusters was determined using the Elbow method (Syakur et al., 2018). We tested k = 2–6; k = 3 explained 72% of the total variance. Cluster robustness was assessed via silhouette scores (>0.6). Based on this criterion, three clusters (k = 3) were selected. The K-means algorithm was then applied to group the Spanish regions into three categories based on their similarity in crime incidence.

### 2.7. Statistical Analysis

Statistical analyses were conducted using SPSS version 29.0 (IBM Corp., Armonk, NY, USA). Results were expressed as absolute frequencies, percentages, and incidence rates per 100,000 inhabitants. A one-way ANOVA with Tukey’s HSD post hoc test was applied to detect regional differences, and correlations between structural variables (education, income, digital access) and offense rates were calculated using Pearson or Spearman coefficients, depending on normality (Shapiro–Wilk test). Regional maps were generated in Microsoft Excel (Office 2016), following ISO 3166 coding standards (ISO—ISO 3166—Country Codes, n.d.).

## 3. Results

### 3.1. National Trends in Cyber-Sex Crimes (2011–2022)

**Figure 1:**
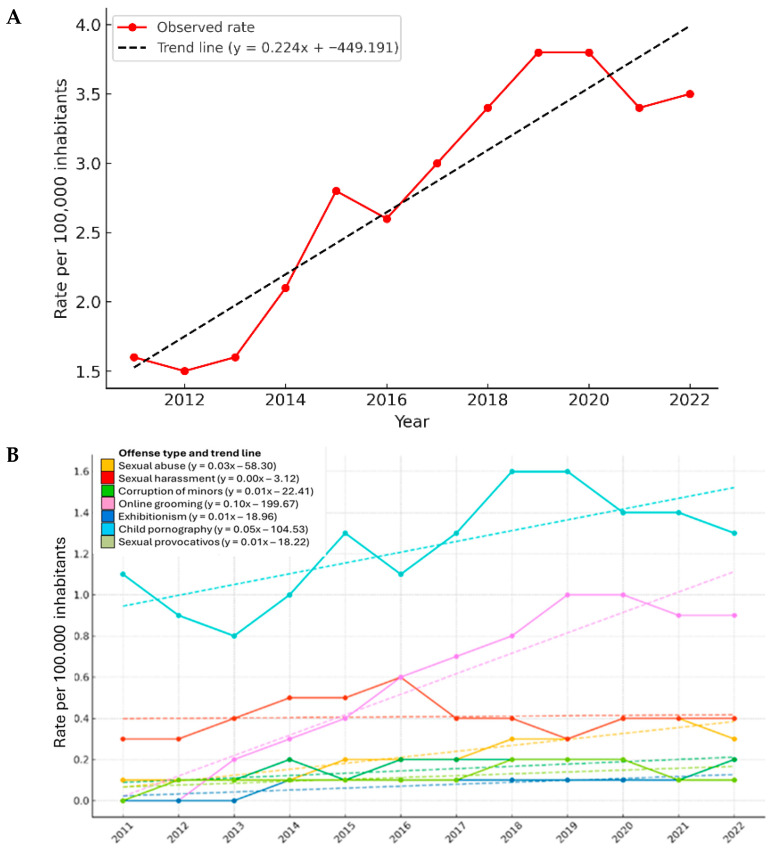
National evolution and linear trends of cyber-sex crime rates in Spain (2011–2022). (**A**) Linear trend in the national incidence rate of cyber-sex crimes in Spain (2011–2022). The figure shows the annual incidence rate per 100,000 inhabitants of all cyber-sex crimes recorded in Spain from 2011 to 2022 (solid red line). A linear regression model was fitted to assess the overall trend during the period (dashed black line). The regression equation is displayed in the legend. The analysis revealed a statistically significant increase over time, with a mean annual growth rate of 0.224 cases per 100,000 inhabitants (R2 = 0.862, *p* < 0.001). (**B**) Linear trends in cyber-sex crime rates by offense type in Spain (2011–2022). The figure displays annual incidence rates per 100,000 inhabitants for seven categories of cyber-sex crimes recorded in Spain between 2011 and 2022. For each offense type, the actual values are represented by solid lines, and the corresponding linear trend lines are shown as dashed lines. The fitted regression line equations are included in the legend to illustrate the direction and magnitude of the trend observed for each category.

The evolution of cyber-sex crime incidence rates in Spain between 2011 and 2022 was analyzed using standardized figures per 100,000 inhabitants (Figure 1A). In 2011, the national rate stood at 1.6 and remained relatively stable during the early years, with a slight decline to 1.5 in 2012 and a return to 1.6 in 2013. From 2014 onwards, a consistent upward trend was observed, with rates rising to 2.1 in 2014 and 2.8 in 2015. After a slight decrease to 2.6 in 2016, the incidence increased again, reaching 3.0 in 2017 and 3.4 in 2018. The peak occurred in 2019, with a rate of 3.8, which remained constant in 2020. In the final years of the series, a slight decrease to 3.4 was recorded in 2021, followed by a modest rebound to 3.5 in 2022. To quantify this trend, a linear regression analysis was performed on the overall national incidence rate. The results indicated a statistically significant increase over the study period, with a mean annual rise of 0.224 cases per 100,000 inhabitants (*p* < 0.001). The model explained 86.2% of the variance (R^2^ = 0.862), confirming a strong and consistent growth in the national rate of reported cyber-sex crimes. In addition to the general trend, the evolution of incidence rates by offense type was also examined (Figure 1B). Child pornography consistently displayed the highest incidence throughout the period. It began at 1.1 in 2011, peaked at 1.6 in 2019 and 2020, and stabilized at 1.3 in 2022. Digital grooming, which had no recorded cases in 2011, showed a marked increase beginning in 2013, reaching 1.0 in 2019 and 2020, and concluding at 0.9 in 2022. Corruption of minors maintained moderate and sustained rates, starting at 0.3 in 2011, peaking at 0.6 in 2015, then fluctuating slightly and ending at 0.4 in 2022. Sexual abuse and sexual harassment followed similar upward patterns, with initial rates around 0.1 in 2011 and maximum values of 0.4 and 0.3, respectively, recorded in 2020. In the final years, these offenses remained stable or declined slightly. Sexual exhibitionism and sexual provocation showed the lowest incidence rates across the entire period, with minimal annual variation. In 2022, exhibitionism and provocation registered rates of 0.2 and 0.1, respectively. The regression analysis by offense type revealed differences in the pace and consistency of growth. Grooming exhibited the steepest increase, with a slope of β = 15.41 cases per year (*p* < 0.001), explaining 91.4% of the variance (R^2^ = 0.914). Sexual harassment (β = 14.14; R^2^ = 0.969; *p* < 0.001) and sexual abuse (β = 12.50; R^2^ = 0.960; *p* < 0.001) also showed significant upward trends. Although child pornography had a more moderate slope (β = 7.27), it presented the highest explanatory value (R^2^ = 0.991), indicating a stable and predictable trajectory over time.

### 3.2. Analysis of Regional Variation and Temporal Progression of Cyber-Sex Crimes in Spain

The evolution of cyber–sex crime rates across Spain’s autonomous communities (CCAA) from 2011 to 2022 revealed apparent regional disparities (Table 1). Andalusia showed a steady rise from 1.5 in 2011 to 4.8 in 2022, particularly between 2015 and 2021. Aragon displayed greater variability, peaking at 4.1 in 2021 before declining to 3.3. The Balearic Islands consistently recorded some of the highest rates, rising from 3.1 in 2011 to 6.3 in 2016, then stabilizing at 5.1 in 2022. Moderate upward trends were observed in Cantabria (up to 3.8 in 2022) and Galicia (peaking at 3.4 in 2020, then slightly declining). Navarre showed a sharp rise from 0.9 in 2011 to 6.5 in 2020, followed by a fall to 3.9 in 2022. La Rioja experienced strong fluctuations, with an exceptional peak of 13.8 in 2020, followed by a drop to 2.5. Ceuta also showed variability, reaching 4.8 in 2022, while Melilla alternated between zero incidence and isolated spikes such as 3.5 in 2017. In Murcia, rates increased steadily from 1.5 to 4.4 in 2019–2020, then decreased to 2.8. Madrid exhibited a stable progression (1.4 to 3.0), with moderate peaks in 2019 and 2021, and the Basque Country reached 5.1 in 2020 before falling to 2.6 in 2022.

### 3.3. Mean Cyber-Sex Crime Rates by Spanish Region (2011–2022)

**Figure 2:**
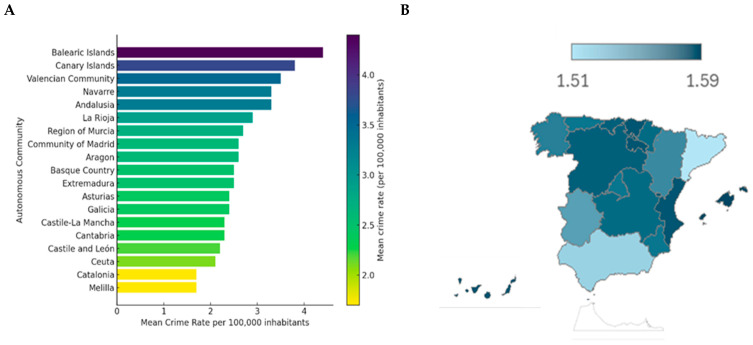
Mean incidence rates of cyber-sex crimes in Spanish regions (2011–2022). (**A**) The mean rate of cyber-sex crimes per 100,000 inhabitants by region. The bar chart displays the mean incidence rate for each autonomous community and city. Notably, the Balearic and Canary Islands show the highest mean rates, followed by Valencian Community, Navarre, and Andalusia. These regions consistently exceeded the national mean throughout the study period. In contrast, Melilla, Catalonia, and Ceuta reported the lowest mean rates. (**B**) Map of Spain by the regional mean rate of cyber-sex crimes. The geographic distribution of rates is visualized using a color scale ranging from light blue (lower values) to dark blue (higher values). This visual representation highlights apparent spatial disparities, with higher concentrations of reported cyber-sex crimes in southern and island regions and lower rates in certain northern and inland areas. The map provides a complementary spatial dimension to the quantitative comparisons shown in the bar chart.

Figure 2 shows the mean cyber–sex crime rates per 100,000 inhabitants across Spain’s autonomous communities, ranked from highest to lowest. Evident regional disparities were observed, likely influenced by tourism intensity, population density, and socioeconomic factors. The Balearic Islands showed the highest mean rate (4.4), followed by the Canary Islands (3.8) and the Valencian Community (3.5), all regions with strong year-round tourism activity. Navarre and Andalusia also reported elevated averages (3.3). Mid-range values were found in La Rioja (2.9), Murcia (2.7), Aragon and Madrid (2.6 each), the Basque Country and Extremadura (2.5), and Asturias and Galicia (2.4). Despite their high urban density, Madrid and the Basque Country did not exhibit the highest rates, possibly reflecting more effective safety policies or reporting differences. The lowest figures were recorded in Castile–La Mancha and Cantabria (2.3), Castile and León (2.2), Ceuta (2.1), and Catalonia and Melilla (1.7).

### 3.4. Spatial Patterns and Cluster-Based Classification of Cyber-Sex Crimes in Spanish Regions

**Figure 3:**
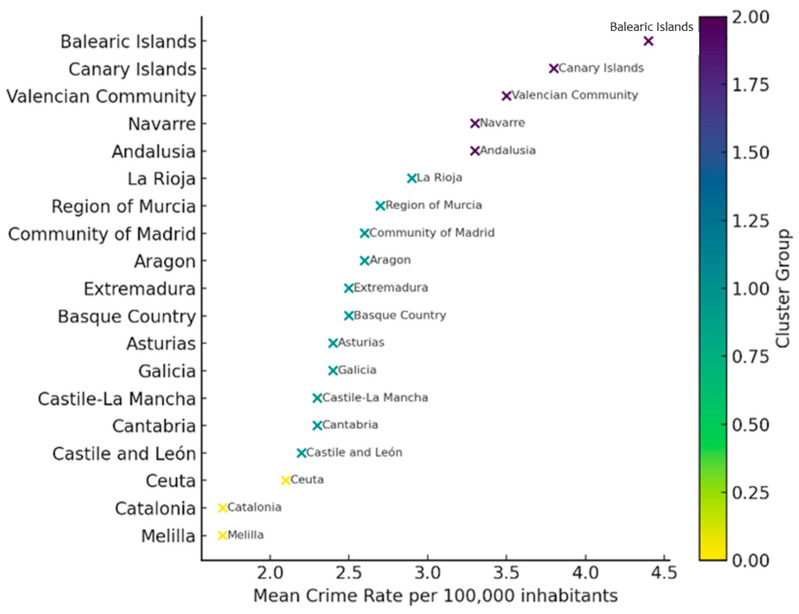
Cluster-based distribution of Spanish regions by mean cyber-sex crime rates (2011–2022). The scatter plot presents a cluster analysis of Spain’s autonomous communities based on their mean cyber-sex crime rates per 100,000 inhabitants between 2011 and 2022. Each point represents a region, positioned according to its mean incidence rate and vertically labeled for identification. Colors indicate cluster membership, as defined by the K-means algorithm: darker shades correspond to the group with the highest crime rates and lighter shades to groups with lower mean rates. The vertical axis is categorical and non-numeric, used solely for regional labeling.

Figure 3 illustrates the spatial distribution of cybersex crime rates in Spain between 2011 and 2022, based on a cluster analysis that identified three distinct groups of autonomous communities according to their mean incidence levels. The classification, shown in a color-coded scatter plot, highlights regional similarities: darker tones denote higher mean rates, while lighter ones correspond to lower incidence. Cluster 0 included the Balearic Islands, the Canary Islands, and Andalusia, regions consistently exceeding the national mean. Cluster 1 comprised communities with intermediate values (Navarre, the Valencian Community, Murcia, Madrid, and Galicia). Cluster 2 grouped those with the lowest mean rates (Catalonia, Aragon, Asturias, Castile and León, Castile-La Mancha, Cantabria, and La Rioja), all showing comparatively lower levels of reported cybersex offenses throughout the study period.

**Figure 4:**
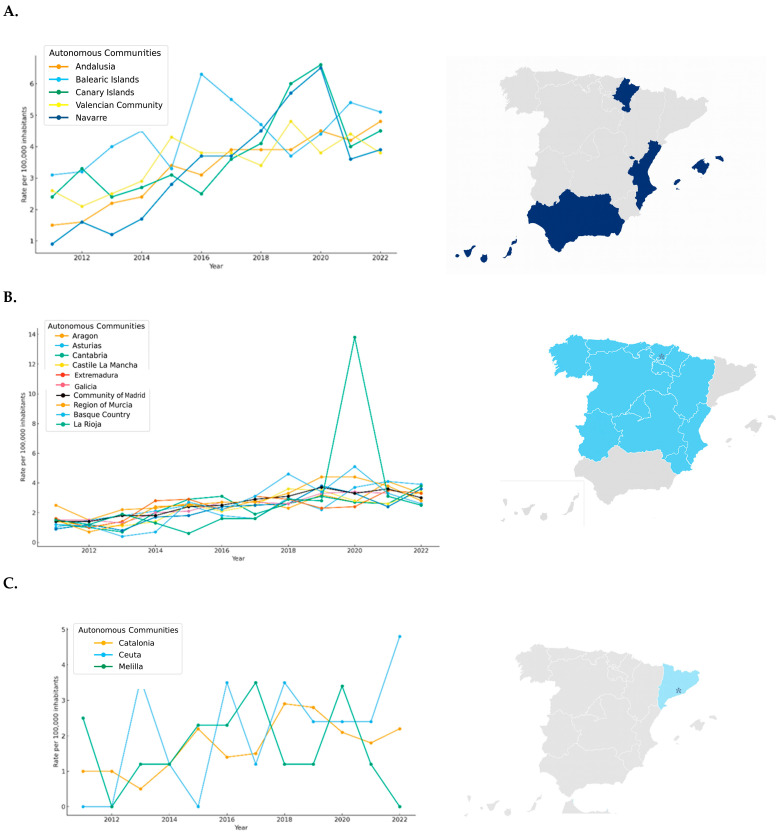
Cluster analysis of cyber-sex crime rates by autonomous community in Spain (2011–2022). Each panel displays the autonomous communities grouped by cluster, according to their mean incidence rate of cyber-sex crimes per 100,000 inhabitants during the study period. The classification highlights regional disparities in reported offenses. * It should be noted that the Mossos d’Esquadra (Catalan Police) and the Ertzaintza (Basque Police) have been reporting data on arrests and investigations only since 2015. Therefore, figures from Catalonia and the Basque Country may be underrepresented. (**A**). Cluster 0-Regions with high mean cyber-sex crime rates, (**B**). Cluster 1-Regions with intermediate mean rates, (**C**). Cluster 2-Regions with low mean rates.

Figure 4 provides a detailed breakdown of the clusters to understand each group’s characteristics better. Cluster 0 (Figure 4A) includes regions with consistently high rates throughout the study period, namely the Balearic Islands, the Canary Islands, and Andalusia, which reported mean values well above the national average and form the upper tier of cybersex crime incidence in Spain. Cluster 1 (Figure 4B) comprises autonomous communities with intermediate rates, such as Murcia, Navarre, the Valencian Community, and Madrid. Although their incidence levels did not reach those of the highest cluster, they remained clearly above the national minimum, representing a stable middle group in terms of crime intensity. Cluster 2 (Figure 4C) includes regions with the lowest incidence levels (Aragon, Asturias, Castile and León, Castile-La Mancha, Galicia, Catalonia, and La Rioja), which consistently recorded lower rates than the rest of the country and occupy the lower range of the national distribution. It should be noted that the Mossos d’Esquadra (Catalan Police) and the Ertzaintza (Basque Police) have reported arrest and investigation data only since 2015; therefore, figures for Catalonia and the Basque Country may be partially underrepresented, and their cluster assignments should be interpreted with caution.

### 3.5. Comparative Analysis of Mean Cyber-Sex Crime Rates by Offense Type and Region

**Figure 5:**
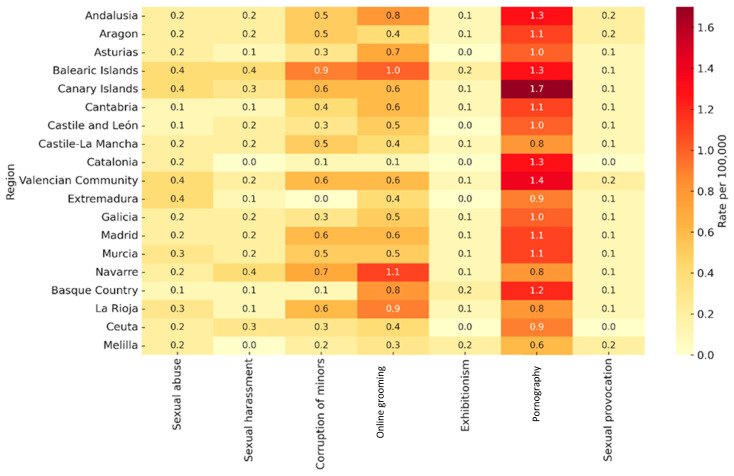
Heatmap of mean annual incidence rates (per 100,000 inhabitants) of seven cyber-sexual crime categories across Spain between 2011 and 2022. Crime types (sexual abuse, sexual harassment, corruption of minors, online grooming, exhibitionism, pornography, and sexual provocation) are shown along the horizontal axis; regions are listed on the vertical axis. Cell color intensity (from pale yellow to deep red) corresponds to the magnitude of the mean rate.

Figure 5 presents the mean rates of cybersex crimes by offense type across Spain’s autonomous communities and cities between 2011 and 2022. The data reveal clear regional patterns, with certain communities consistently showing higher rates across multiple categories. Child pornography emerged as the offense with the highest national mean, particularly in the Canary Islands (1.7), the Valencian Community (1.4), Catalonia (1.3), and Andalusia (1.3), where it accounted for a large share of overall cybersexual offenses. Several regions, including the Balearic Islands, Navarre, and Andalusia, displayed elevated mean rates in multiple categories. The Balearic Islands reported some of the highest figures in online grooming (1.0), sexual harassment (0.4), and corruption of minors (0.9), while Navarre led in grooming (1.1). Andalusia consistently exceeded national means across nearly all offense types. In contrast, regions such as Catalonia, Castile and León, Asturias, and Galicia maintained notably low figures for harassment, exhibitionism, and sexual provocation, although Catalonia showed comparatively high rates for child pornography. Despite their smaller populations, Ceuta and Melilla also recorded particularly high values for certain offenses: Ceuta in child pornography (0.9) and Melilla in exhibitionism and sexual provocation (0.2 each), occasionally surpassing larger regions.

### 3.6. Analysis of Correlations Between Sociodemographic, Educational, Economic, and Technological Variables and the Incidence of Cyber-Sexual Crimes (2022)

**Figure 6:**
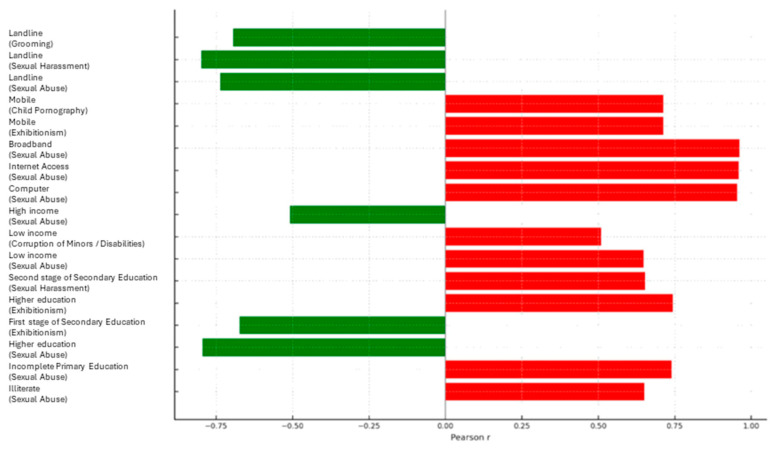
Significant correlations between structural factors and cyber-sexual crimes (Spain, 2022). Horizontal bar chart showing significant Pearson correlations (*p* < 0.05) between educational level, income, and internet access indicators and the incidence of various types of cyber-sexual crimes per 100,000 inhabitants in Spain. Positive correlations (↑) are shown in red; negative correlations (↓) in green.

Table 2 and Figure 6 show statistically significant associations between various sociodemographic, educational, economic, and technological variables and the rates of cybersexual crimes recorded in Spain in 2022. These associations are based on aggregate regional data and should be interpreted at the ecological level, without implying individual-level causality. Regarding educational level, a significant correlation was observed between illiteracy and sexual abuse (r = 0.650, *p* = 0.030), and between incomplete primary education and sexual abuse (r = 0.739, *p* = 0.009) (Figure 4). Higher education showed a significant negative correlation with sexual abuse (r = −0.795, *p* = 0.003) and a positive correlation with exhibitionism (r = 0.743, *p* = 0.008). Similarly, the second stage of secondary education showed a significant correlation with sexual harassment (r = 0.652, *p* = 0.029). On the other hand, income data show that low income is significantly associated with sexual abuse (r = 0.648, *p* = 0.007) and corruption of minors (r = 0.509, *p* = 0.044), while high income shows a negative correlation with sexual abuse (r = −0.508, *p* = 0.044). Finally, in the technological field, access to computers, the Internet, and broadband are significantly associated with all forms of cyber-sexual crimes, with the most notable being grooming (r = 0.956 to 0.972, *p* < 0.001), exhibitionism (r = 0.773 to 0.800, *p* = 0.001–0.003), and child pornography (r = 0.748 to 0.762, *p* = 0.004–0.005). Negative correlations are also observed between landline use and several offenses, such as sexual abuse (r = −0.737, *p* = 0.006) and grooming (r = −0.695, *p* = 0.012). Mobile phone use shows significant positive correlations with grooming (r = 0.939, *p* < 0.001), exhibitionism (r = 0.712, *p* = 0.009), and child pornography (r = 0.712, *p* = 0.009).

## 4. Discussion

This study aimed to identify structural and contextual factors associated with the incidence of cyber-sexual crimes in Spain by examining the relationships between reported offense rates and a range of educational, economic, and technological indicators at the regional level.

### 4.1. Structural and Regional Inequalities

The findings confirm marked territorial disparities in the prevalence of cyber-sexual crimes. Southern and island regions such as the Balearic and Canary Islands and Andalusia consistently recorded the highest rates, while communities such as Galicia, Castilla-La Mancha, and Catalonia reported significantly lower levels. These differences reflect diverse socioeconomic and digital contexts mediating vulnerability across regions. The results are consistent with a UNICEF (2021) report (Fuentes et al., n.d.) highlighting how structural inequalities (such as child poverty, educational deficits, and digital exclusion) are unevenly distributed across Spanish autonomous communities, influencing children’s exposure to online risks and their capacity to access protection.

Regions with a higher share of residents lacking formal education or having only incomplete primary education were associated with higher reported rates of sexual abuse, although causality cannot be inferred from these aggregated data. This suggests that educational background may shape digital behavior patterns and risk awareness. The “Silenciadas” report by Save the Children (2024) (Informe “Silenciadas” | Save the Children, n.d.) stresses the importance of comprehensive sexual education in preventing sexual violence among adolescents and reducing distorted perceptions of sexuality and consent, particularly in socially disadvantaged areas.

Economic vulnerability also revealed consistent associations. Low-income regions were significantly linked to higher rates of sexual abuse and corruption of minors, while higher-income areas showed negative correlations with these same crimes. These findings support the view that economic disadvantage creates environments with elevated risk exposure and weaker institutional resources. The review by Espinosa Zárate et al. (2023) on digitalization in vulnerable populations reinforces this idea, showing that digital initiatives often fail when structural inequalities are not addressed. Similarly, studies on digital exclusion (Ragnedda et al., 2022) and adverse digital incorporation (Heeks, 2021) highlight how unequal access to and use of technology can perpetuate cycles of marginalization. It is worth noting, however, that the association between socioeconomic disadvantages and higher prevalence of online victimization does not appear uniformly across all types of cybercrime. While our findings confirm that poverty is consistently linked to greater exposure to cyber-sexual crimes, this pattern is not evident in other forms of cyber offending. This specificity suggests that structural inequalities may play a particularly critical role in shaping vulnerability to online sexual harm, reinforcing the need to address socioeconomic disadvantage as a central component of prevention strategies in this area.

### 4.2. Technological Factors and Emerging Digital Risks

On the other hand, technological factors also emerged as key explanatory variables. Higher levels of mobile phone use, broadband access, and computer ownership were positively associated with offenses such as grooming, child pornography, and exhibitionism. This pattern may partly reflect better reporting infrastructure in more connected regions rather than higher perpetration rates. In contrast, landline usage was negatively correlated with these crimes. However, differences in demography (average age, urbanization) could confound this negative relationship and warrant multivariate control in future analyses. These patterns suggest that it is not digital connectivity per se that increases risk, but the type and intensity of digital engagement. Regions with broader access to fast, personal, and mobile digital tools may offer greater opportunities for exposure to harmful content and for perpetrating offenses.

At first sight, the association between broader digital access and higher prevalence of cyber-sexual crimes might appear self-evident. However, our results indicate that this relationship is not uniform across all forms of technology. While access to personal and mobile digital tools such as computers, broadband, and smartphones is positively correlated with offenses like grooming or pornography, more traditional infrastructures such as landline telephony display the opposite association. This finding suggests that it is not connectivity per se that increases exposure, but rather the type of technological resources available and the intensity of their use, which shape distinct patterns of regional vulnerability. Another relevant dimension relates to the role of poor cybersecurity practices and deficient “cyber hygiene” as enabling factors for cyber-sexual victimization. As Choi and Lee (2017) noted, low awareness of digital risks, inadequate password management, unsafe browsing behaviors, and limited training in protective measures significantly increase individual vulnerability to online threats. These weaknesses create opportunities for offenders to exploit technical and behavioral gaps, facilitating grooming, image-based abuse, and other forms of cybercrime. In this sense, strengthening digital literacy and promoting basic cybersecurity practices should be considered essential components of prevention strategies, complementing structural, socioeconomic, and technological factors already identified in this study.

A recent study emphasizes that digital environments pose specific risks to mental autonomy, cognitive liberty, privacy, and integrity, concerns now framed within the broader debate on neurorights (Segate, 2022). These issues go beyond traditional notions of harm and invite reflection on how online experiences may affect cognitive processes differently from offline ones. Although existing frameworks do not yet draw clear boundaries between both spheres, they stress the importance of recognizing digital assaults on the mind as distinctive forms of victimization. From this perspective, law enforcement may need to adapt its strategies to address cognitive harm in online contexts, in line with the evolving discourse on neurorights. It should also be acknowledged that the observed increase in cyber-sexual crimes may reflect two overlapping dynamics: on the one hand, the genuine expansion of harmful practices facilitated by digital environments, and on the other, the greater visibility and categorization of behaviors that may have long existed offline but were less frequently reported or socially problematized. This limitation highlights the difficulty of disentangling new forms of online victimization from the reconfiguration of pre-existing practices within the digital sphere. Although our analysis is limited to officially recorded cases of cyber-sexual crimes, it is important to acknowledge emerging concerns around sexual harassment in immersive digital environments such as virtual and mixed reality. As recent scholarship highlights (Rydzewski, 2024), these technologies create new spaces for interaction where existing forms of abuse can be replicated or intensified, often with blurred boundaries between “virtual” and “real” harm. While such incidents are not yet systematically captured in police databases in Spain, they represent a frontier of cyber-victimization that warrants scholarly and policy attention. Future research should therefore consider how these environments may shape new modalities of sexual harassment and whether current legal and law enforcement frameworks are adequately prepared to address them.

### 4.3. Spatial Patterns, Prevention Strategies, and Limitations

Finally, the geographic clustering of regions with high, medium, and low crime rates confirms that cyber-sexual victimization is not randomly distributed but follows structural and spatial logic. This reinforces the need for territorial prevention strategies considering localized vulnerabilities, including socioeconomic inequality, educational disadvantages, and digital infrastructure gaps.

Overall, this study contributes not only empirical evidence on regional disparities in cyber-sexual crimes but also a conceptual perspective linking these patterns to broader structural and technological inequalities.

This study is subject to several limitations. First, the analysis relied on officially recorded data from the Spanish Ministry of the Interior. As such, it excludes unreported incidents, which may result in an underestimation of the true prevalence of cyber-sexual crimes. This is a common challenge in cybercrime research, particularly for offenses involving vulnerable populations. Furthermore, aggregated data preclude inferences about individual risk factors, exposing the analysis to ecological fallacy. With only 19 regions in 2022, correlational analyses are underpowered; future work should exploit the full 2011–2022 panel using multilevel models with year-fixed effects to improve robustness. Second, due to limitations in regional data submission, particularly from Catalonia and the Basque Country (where official reporting by regional police forces began only in 2015), these areas may be partially underrepresented. This affects the accuracy of historical comparisons and the interpretation of cluster assignments. Finally, the data were aggregated at the regional level, limiting the ability to explore individual or household-level dynamics. Despite this, the analysis provides valuable insights into how broader structural factors (such as education, income, and digital access) relate to territorial differences in cyber-sexual victimization.

## 5. Conclusions

This study identifies marked regional disparities in cyber-sexual crimes in Spain between 2011 and 2022. The Balearic Islands, Canary Islands, and Andalusia consistently showed the highest rates, reflecting structural vulnerabilities linked to socioeconomic inequality and patterns of digital access. Lower educational attainment and income were significantly associated with higher prevalence of sexual abuse and corruption of minors, while broader access to mobile phones, broadband, and computers correlated with grooming, pornography, and exhibitionism. In contrast, traditional infrastructures such as landlines showed negative associations, pointing to differing digital usage profiles across regions. These results confirm that cyber-sexual victimization is shaped not only by individual behaviors but also by structural and technological conditions. Effective prevention requires territorially sensitive strategies that combine equitable education, socioeconomic support, and stronger digital literacy and cybersecurity practices. Such approaches are essential to reduce regional vulnerabilities and improve protection against technology-facilitated sexual crimes.

## Figures and Tables

**Table 1:** Evolution of cyber-sex crime rates across Spanish regions from 2011 to 2022.

Regions	2011	2012	2013	2014	2015	2016	2017	2018	2019	2020	2021	2022
Andalusia	1.5	1.6	2.2	2.4	3.4	3.1	3.9	3.9	3.9	4.5	4.2	4.8
Aragon	2.5	1.5	2.2	2.3	2.6	2.2	2.8	2.3	3.2	2.7	4.1	3.3
Asturias	1.0	1.5	1.8	2.1	2.5	1.8	1.6	3.0	2.2	3.7	4.1	3.9
Balearic Islands	3.1	3.2	4.0	4.5	3.3	6.3	5.5	4.7	3.7	4.4	5.4	5.1
Canary Islands	2.4	3.3	2.4	2.7	3.1	2.5	3.6	4.1	6.0	6.6	4.0	4.5
Cantabria	1.2	1.0	0.7	2.0	2.9	3.1	1.9	2.6	3.1	2.7	2.6	3.8
Castile and Leon	0.9	1.2	0.8	1.7	1.8	2.4	2.5	2.6	3.8	3.3	2.4	3.6
Castile-La Mancha	1.4	1.1	1.1	1.4	2.5	2.1	2.5	3.6	3.5	2.8	2.6	3.4
Catalonia	1.0	1.0	0.5	1.2	2.2	1.4	1.5	2.9	2.8	2.1	1.8	2.2
Valencian Community	2.6	2.1	2.5	2.9	4.3	3.8	3.8	3.4	4.8	3.8	4.4	3.8
Extremadura	1.6	1.0	1.4	2.8	2.9	2.2	3.1	2.9	2.3	2.4	3.5	3.3
Galicia	1.5	1.5	1.3	2.0	2.1	2.7	2.7	2.6	3.3	3.4	3.3	2.6
Madrid	1.4	1.4	1.8	1.8	2.4	2.5	2.9	3.1	3.7	3.3	3.6	3.0
Murcia	1.5	0.7	1.2	2.4	2.5	2.7	2.7	3.3	4.4	4.4	3.8	2.8
Navarre	0.9	1.6	1.2	1.7	2.8	3.7	3.7	4.5	5.7	6.5	3.6	3.9
Basque Country	1.2	1.1	0.4	0.7	2.7	2.2	3.1	4.6	3.4	5.1	3.3	2.6
Rioja	1.5	1.2	1.9	1.3	0.6	1.6	1.6	2.9	2.8	13.8	3.1	2.5
Ceuta	0.0	0.0	3.6	1.2	0.0	3.5	1.2	3.5	2.4	2.4	2.4	4.8
Melilla	2.5	0.0	1.2	1.2	2.3	2.3	3.5	1.2	1.2	3.4	1.2	0.0

Rates are expressed per 100,000 inhabitants. Regions correspond to Spain’s autonomous communities.

**Table 2:** Analysis of correlations between sociodemographic, educational, economic, and technological variables and the incidence of cyber-sexual crimes (2022).

Types of Cyber-Sexual Crimes	Sexual Abuse	Sexual Harassment	Corruption of Minors/Disabilities	Grooming	Exhibitionism	Child Pornography	Sexual Provocation
A. Educational level							
Illiterate	0.650 *	0.111	0.233	0.185	−0.310	−0.193	0.497
Incomplete Primary Education	0.739 **	0.010	0.033	0.233	−0.288	−0.468	0.226
Primary Education	0.006	0.021	0.142	−0.217	−0.195	0.022	−0.171
First stage of Secondary Education	0.600	0.079	0.243	−0.074	−0.673 *	−0.073	0.158
Second stage of Secondary Education	0.025	0.652 *	0.407	−0.011	0.016	0.296	−0.005
Secondary education, vocational training	−0.398	0.009	0.108	0.082	0.164	0.236	0.206
Higher education	−0.795 **	−0.351	−0.481	0.020	0.743 **	0.093	−0.256
B. Mean annual net income per inhabitant							
Low	0.648 **	−0.180	0.509 *	−0.083	−0.246	−0.017	0.338
Lower middle	−0.031	0.004	−0.148	−0.294	−0.125	0.304	0.011
Upper middle	−0.366	0.361	−0.365	0.017	−0.173	0.180	−0.066
High	0.508 **	−0.014	−0.278	0.261	0.462	−0.271	−0.358
C. Internet and social media usage rate							
Ever used the Internet	−0.154	0.130	−0.084	−0.217	0.062	−0.176	−0.063
Use in the last 12 months	−0.149	0.109	0.008	−0.262	0.031	−0.073	−0.145
Use in the last 3 months	−0.21	0.136	0.005	−0.114	0.111	0.045	−0.166
Weekly use	−0.176	0.078	−0.019	−0.153	0.000	−0.033	−0.085
Daily use	0.011	0.338	−0.060	−0.106	0.044	−0.045	0.025
Multiple daily uses	0.065	0.214	0.016	−0.181	0.086	−0.259	−0.081
D. Type of Internet access equipment and computer connections							
Computer	0.954 ***	0.931 ***	0.189	0.956 ***	0.773 **	0.748 **	0.501
Internet Access	0.959 ***	0.936 ***	0.255	0.972 ***	0.800 ***	0.762 **	0.530
Broadband	0.961 ***	0.936 ***	0.249	0.972 ***	0.794 **	0.760 **	0.528
Landline	−0.737 **	−0.798 ***	−0.076	−0.695 **	−0.438	−0.434	−0.536
Mobile	0.960 ***	0.926 ***	0.225	0.939 ***	0.712 **	0.712 **	0.575

Values represent Pearson correlation coefficients (r) and *p*-values between variables related to internet use, educational attainment, and technological access, as well as the rates of cyber-sexual offenses per 100,000 inhabitants. Statistical significance (*p* < 0.05) was considered. * *p* < 0.05; ** *p* < 0.01; *** *p* < 0.001.

## Data Availability

https://estadisticasdecriminalidad.ses.mir.es/publico/portalestadistico/en/.a (accessed on 20 February 2025).
